# Effect of TiC Content on Microstructure and Wear Performance of 17-4PH Stainless Steel Composites Manufactured by Indirect Metal 3D Printing

**DOI:** 10.3390/ma16196449

**Published:** 2023-09-28

**Authors:** Xiao Huang, Shuo Mei, Yazhi Li, Mingyang Li, Shujun Zhou, Hongfei Shang

**Affiliations:** 1 School of Mechanical and Electrical Engineering, China University of Mining and Technology (Beijing), Beijing 100083, China; hx@cumtb.edu.cn (X.H.); meishuo2023@163.com (S.M.); liyazhi1999@163.com (Y.L.); lmy1353322604@163.com (M.L.); 2 State Key Laboratory of Tribology, Tsinghua University, Beijing 100084, China; shanghongfei@tsinghua.edu.cn

**Keywords:** 3D printing, fused filament fabrication, TiC, 17-4PH stainless steel, microstructure, wear resistance

## Abstract

In order to improve the performance of 17-4PH under wear conditions (e.g., gears, etc.) and reduce the cost of metal additive manufacturing, TiC needs to be added to 17-4PH to improve its wear resistance. Micron-sized TiC-reinforced 17-4PH stainless steel composites with different contents (0–15 wt%) have been prepared by fused filament fabrication 3D printing for the first time. The effects of TiC content on the structure and properties of composites were studied by XRD, SEM, and sliding wear. The obtained results show that the microstructure of TiC-reinforced 17-4PH stainless steel composites mainly consists of austenite. With the increase in TiC content, the grain size is obviously refined, and the average grain size decreases from 65.58 μm to 19.41 μm. The relative densities of the composites are maintained above 95% with the addition of TiC. The interfaces between TiC particles and the 17-4PH matrix are metallurgical bonds. The hardness of the composites increases and then decreases with increasing TiC content, and the maximum hardness (434 HV) is obtained after adding 10 wt.% of TiC content. The wear rate of the composites was reduced from 2.191 × 10^−5^ mm^3^ /(N‧m) to 0.509 × 10^−5^ mm^3^ /(N‧m), which is a 3.3-fold increase in wear resistance. The COF value declines with the addition of TiC. The reasons for the significant improvement in the composites’ performance are fine grain strengthening, solid solution strengthening, and second phase strengthening. The wear mechanisms are mainly abrasive and adhesive wear. Compared to the 10 wt% TiC composites, the 15 wt% TiC composites show limited improvement in wear resistance due to more microcracks and TiC agglomeration.

## 1. Introduction

Metal fused filament fabrication (FFF) is a new metal additive manufacturing technology that combines polymer fused deposition and metal powder injection [1,2,3,4]. The process consists of metal powder and polymer composite filaments printed layer by layer on a 3D printer to make green printed parts, which are degreased and sintered to obtain fully dense metal objects [4,5,6]. Compared to other metal fabrication techniques (casting, metal powder injection, and machining), this technology has the advantages of low cost, no need for molds, no material loss, etc. [7,8]. This process has become increasingly popular for various material fabrications, such as titanium alloys, pure copper, Ni-based alloys, cemented carbides, Al alloys, and stainless steel [9]. Additionally, 17-4PH stainless steel is commonly used in metal additive manufacturing and has the advantages of a cheap price, good mechanical properties, corrosion resistance, etc. It is widely used in the aerospace, mold, medical, automotive, and energy industries [1,10,11]. With the increasing complexity of the application environment, ceramic-reinforced metal composites have become a developing need. The addition of ceramic particles to 17-4PH stainless steel can greatly boost its strength and wear resistance, expanding its range of applications. Commonly used ceramic particles are TiC, TiN, SiC, WC, etc. [12,13,14,15,16]. Among these hard ceramic particles, TiC has excellent physical and chemical properties such as a high melting point, high hardness, low density, wear resistance, and thermal stability. In particular, the wetting angle θ between TiC particles and liquid Fe is less than 50°, which will facilitate the formation of a good interfacial bond between TiC and Fe matrix during sintering [17]. Therefore, TiC is very suitable to be used as a strengthening phase for steels [18]. In recent years, the addition of TiC particles to reinforced iron-based composites has gradually become a research hotspot because its mechanical properties and wear resistance can be significantly improved. Onuoha et al. [19] prepared TiC-316L cermets with different TiC particle sizes by melt infiltration method to study the effect of TiC particle size on the dry reciprocating wear behavior of stainless steel cermets. The results showed that small-sized TiC particles had better wear resistance and hardness compared to large-sized TiC particles. The wear rate was positively correlated with the normal load and the metal phase content. Wanli Ma et al. [20] prepared 1Cr12Ni3Mo2V composite specimens reinforced with different contents of TiC particles based on laser solid forming technology, and the microstructure analysis and friction and wear properties of each specimen were tested. The results showed that TiC particles were well bonded to the interface of 1Cr12Ni3Mo2V stainless steel. The hardness and wear resistance were significantly improved. Akhtar Farid et al. [21] prepared particulate TiC-reinforced 465 maraging stainless steels matrix composites using a conventional powder metallurgy process and evaluated the microstructure, mechanical properties, and wear properties of the composites. The findings demonstrated that the TiC particles adhered well to the 465 stainless steel matrix phase and that tensile stresses induced during sintering caused microcracks in the composites. The martensitic stainless steel’s heat treatment procedure, TiC content, and particle spacing all had a significant impact on the composites’ wear characteristics. All of the above studies have shown that TiC iron-based composites have significantly improved strength and wear resistance compared to matrix steel. With the development of science and technology, people have put forward new demands for the lightweight characteristics of mechanical parts. While meeting the requirements of mechanical properties of parts (such as lightweight gears), determining how to minimize their quality has become an urgent engineering problem. The existing lightweight technology mainly includes structural optimization and material optimization. With the rapid development of metal additive manufacturing technology, lightweight parts can be mass produced [22,23].

At present, the development of high-performance TiC iron-based composites by selective laser melting (SLM) technology has been extensively investigated [24,25,26], but there is little research on the metal fused deposition molding process for TiC iron-based composites. Among several technologies for metal additive manufacturing, FFF utilizes low-cost equipment with simplicity and safety. As compared to other common metal additive manufacturing processes such as selection laser melting (SLM) and wire and arc additive manufacturing (WAAM), it does not require loose metal powder or a high-power source [27]. Pure Cu is highly reflective, conductive, and thermally conductive, which makes it difficult to fabricate high-quality Cu parts using conventional techniques such as selective laser melting (SLM) and electron beam melting (EBM). In addition, the preparation of cemented carbides using SLM technology is prone to cracking defects and usually requires subsequent treatments such as elemental fusion infiltration to improve the overall performance. In contrast, cemented carbide components prepared by sintering-based technical routes tend to be free of visible cracks and have excellent mechanical properties with uniform material organization and distribution [28,29,30,31]. On the other hand, most TiC-particle-reinforced metal matrices are aluminum-based, iron-based, and nickel-based alloys; however, the strengthening mechanism of 17-4PH maraging stainless steels has not been extensively studied.

In this paper, in order to prepare lightweight mechanical parts (gears for aerospace) with excellent wear resistance, TiC-reinforced 17-4PH stainless steel composites were prepared for the first time by using metal fused filament fabrication 3D printing technology, and the effects of adding micron-sized TiC with different contents (0, 3, 5, 10, 15 wt%) on the microstructure, hardness, and wear resistance of 17-4PH stainless steel were investigated. The phase composition, densification, and interfacial bonding behavior of the composites were analyzed. This study can provide a reference for the application of FFF technology in the preparation of metal wear-resistant materials.

## 2. Materials and Methods

### 2.1. Materials

**Figure 1:**
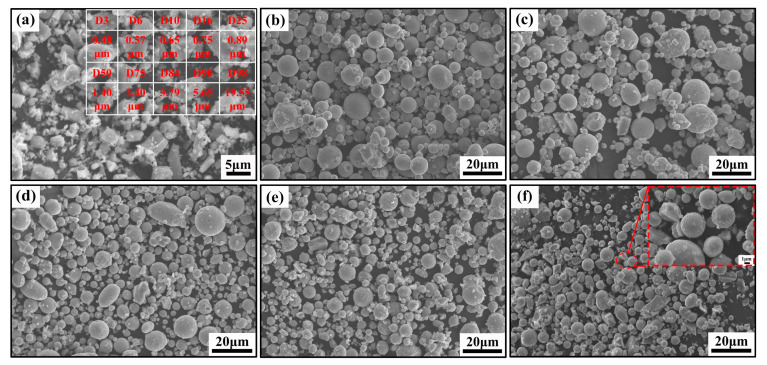
SEM micrographs of powders: (**a**) TiC powder and particle size distribution; (**b**) 17-4PH stainless steel powder; (**c**) 17-4PH + 3 wt% TiC; (**d**) 17-4PH + 5 wt% TiC; (**e**) 17-4PH + 10 wt% TiC; (**f**) 17-4PH + 15 wt% TiC.

The TiC particles were 99.9% pure (D_50_ = 1.40 μm). The particle size of 17-4PH powder was 600-grit. The components of 17-4PH stainless steel are shown in Table 1. Using the two powders as raw material, 3%, 5%, 10%, and 15 wt% of TiC were mixed with 17-4PH stainless steel powder through a 3D powder mixer. The mixer speed was 33 rpm/min, and the mixing duration was 2 h. Figure 1 displays SEM images of TiC, 17-4PH stainless steel powders, and mixed powders. In this experiment, the binder system included 82 wt% polyoxymethylene (POM), 5 wt% high-density polyethylene (HDPE), 3 wt% ethylene vinyl acetate copolymer (EVA), 5 wt% ethylene bis stearamide (EBS), 3 wt% pe wax (PW), and 2 wt% stearic acid (SA). As shown in Figure 1a, the particle size distribution of TiC powder is not uniform. It is mainly composed of small particles and a small amount of larger debris. Figure 1b shows that the 17-4PH stainless steel powder is spherical in shape. The TiC particles are uniformly dispersed throughout the powder mixture in Figure 1c–f, and the small TiC particles are uniformly bonded to the stainless steel particles.

### 2.2. Sample Preparation

**Figure 2:**
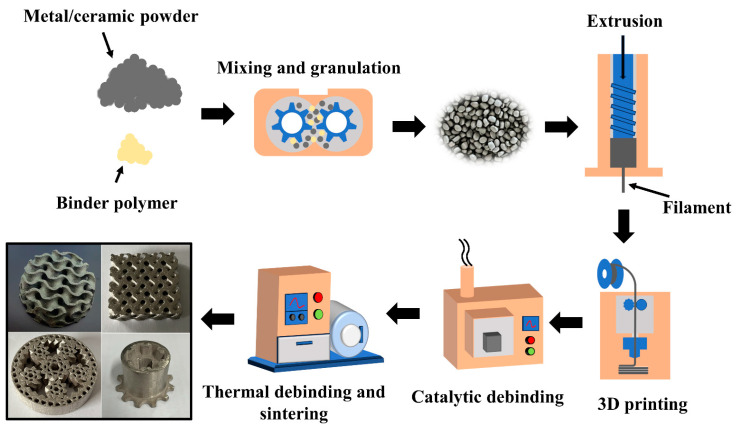
Metal FFF process flow.

Figure 2 shows the process flow of metal fused filament fabrication. First, the metal-ceramic powder was mixed with the binder and pelletized by an integrated mixing and pelletizing machine (MH-10L-DCSS-H, Genyilong Machinery Equipment Co., Ltd., Qingdao, China). The mixing and pelletizing machine applied a three-screw working mode (two forward-rotating and one reverse-rotating), which ensured uniformity in the mixing of metal-ceramic powders and binders. Following pelletization, the pellets were heated to 170 °C in a filament extruder (FLD-25A, Flanders Machinery Co., Ltd., Zhangjiagang, China) and extruded through a screw to produce 3D-printed filaments with a diameter of 1.75 mm ± 0.03 mm. The filaments were printed as 3D-printed samples by an FFF printer (Pro2, Raise3D, Shanghai, China), and Table 2 shows the parameters of the 3D printing process.

**Figure 3:**
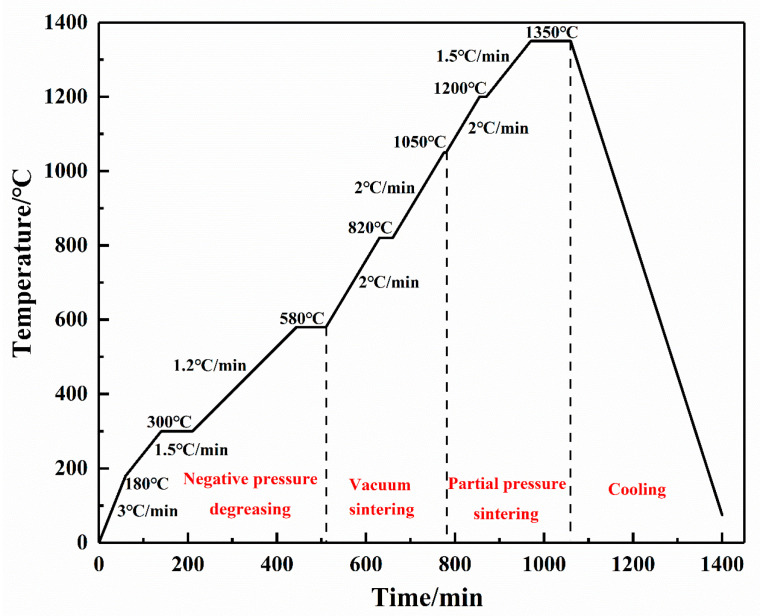
Thermal degreasing and sintering process.

The 3D-printed samples were degreased from paraformaldehyde by oxalic acid- catalyzed degreasing oven (CD-2200A, Sibairui Automatic Control Equipment Co., Ltd., Ningbo, China) at a degreasing temperature of 120 °C and a degreasing time of 8h. Thermal degreasing and sintering of the degreased specimens were carried out in an atmospheric sintering furnace (Hechuan Technology Co., Ltd., Wuxi, China). Figure 3 shows the thermal degreasing and sintering process.

### 2.3. Microstructure and Mechanical Properties Tests

The surface morphology and low-magnification microstructures were observed by an optical microscope (OM, ICX41M, Sunny Optical Technology(group) Co., Ltd., Ningbo, China). The density of the sintered specimens was measured by Archimedes drainage method and the densities of the specimens were calculated. Scanning electron microscope (SEM, JSM-7800F, JEOL Ltd., Tokyo, Japan) and X-ray energy dispersive spectroscopy (EDS, X-MaxN50, Oxford Instruments Technology Co., Oxford, UK,) were used to observe the microstructure of the specimens, the distribution of the elements, and the morphology of the wear surface. The phase composition of the sintered specimens with different TiC contents was characterized by an X-ray diffractometer (XRD, Rigaku SmartLab SE, Rigaku Co., Tokyo, Japan) with Cu-K_α1_ radiation. The scanning range was 30°–90°. The universal hardness tester (TH903, Beijing Shidai Zhifeng Technology Co., Ltd., Beijing, China) was used to measure the hardness of the sample under a load of 100 kg. Each sample was measured five times. The wear resistance was evaluated by the WTM-2E friction and wear tester at room temperature. The normal load was 6 N, the sliding time was 60 min, the rotational speed was 500 r/min, and the wear radius was 3 mm. The Si_3_N_4_ ball with a diameter of 5 mm was selected as a counterpart. The maximum contact pressure was 1.02 GPa. The 3D morphology images of the wear track were measured by a three-dimensional white light interferometer (ZYGO Nexview, Zygo Co., Middlefield, CT, USA), and meanwhile, the wear volume was derived.

## 3. Results and Discussion

### 3.1. Phases Analysis

**Figure 4:**
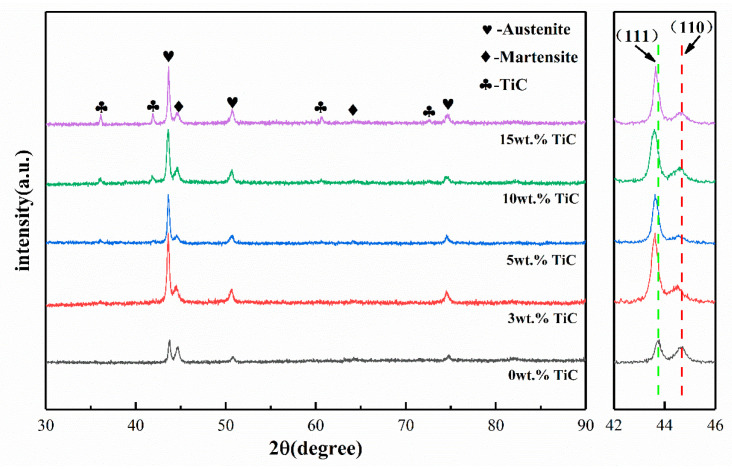
XRD patterns of TiC-17-4PH composite materials with different TiC contents.

Figure 4 shows the X-ray diffraction patterns of TiC-17-4PH composites with different TiC contents. Without the addition of TiC particles, the XRD diffraction peaks mainly consist of austenite and martensite phases, and the diffraction peaks intensities do not differ much. The intensity of the martensitic phase XRD diffraction peaks decreases with increasing TiC content in comparison to the austenitic phase. The main reason for the decrease in martensite content may be the uniform dispersion and partial dissolution of TiC powder within the matrix, where element C can act as a strong austenite stabilizer to maintain the austenite organization up to room temperature [32], and therefore, the relative content of austenite phase increases significantly with the addition of TiC powder. Due to the low TiC content and the decomposition of partial TiC during high-temperature sintering, only austenitic and martensitic phases were observed in the diffraction peaks of 3 wt% TiC, and almost no TiC phase was detected.

It is worth noting that the (111) and (110) peaks of the TiC-reinforced composites obviously shift to the lower angle in comparison with that of the 17-4PH stainless steel without the TiC addition. According to Brag’s law, the expanded space between neighboring lattice planes might be the cause of a drop in the 2θ value, so this phenomenon is attributed to the solid solution effect of Ti atoms. During the partial decomposition of TiC particles during high-temperature sintering, the atomic radius of Ti (1.47 Å) is larger than that of Fe (1.27 Å), so the solid solution of decomposed Ti atoms extends the lattice structure, which increases the face-to-face spacing of the austenite and martensite phases, causing the peaks to move in the direction of the low diffraction angle instead of the high diffraction angle [33,34].

### 3.2. Microstructural Analysis

**Figure 5:**
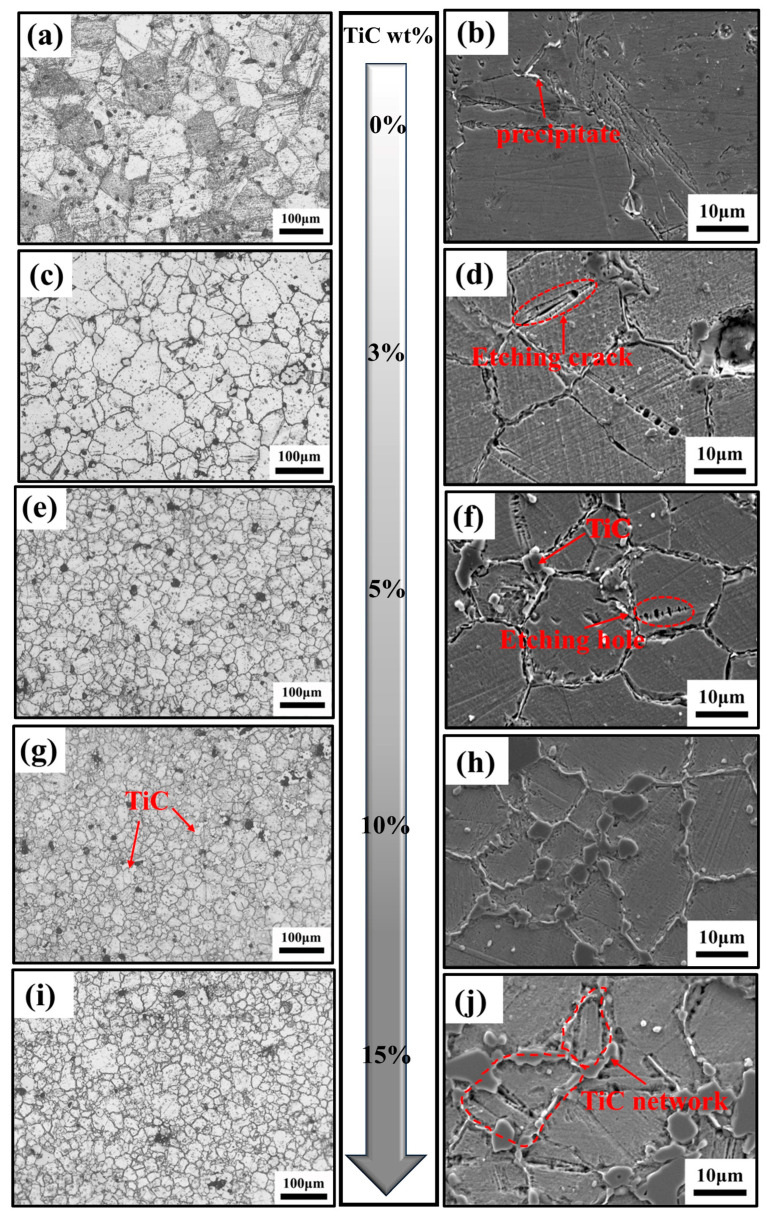
Surface morphology of the sample after etching: (**a**,**b**) 17-4PH +0 wt% TiC; (**c**,**d**) 17-4PH + 3 wt% TiC; (**e**,**f**) 17-4PH + 5 wt% TiC; (**g**,**h**) 17-4PH + 10 wt% TiC; (**i**,**j**) 17-4PH + 15 wt% TiC.

**Figure 6:**
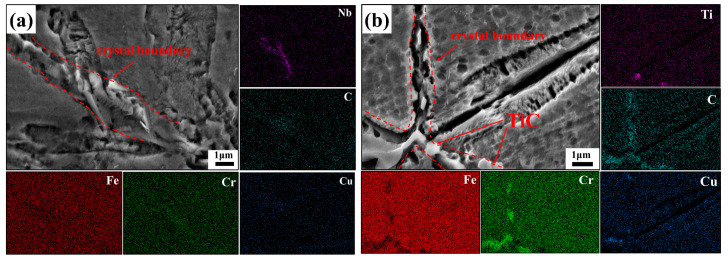
EDS spectrum of intergranular precipitates: (**a**) 17-4PH + 0 wt% TiC; (**b**) 17-4PH + 3 wt% TiC.

Figure 5 depicts the metallographic organization of sintered specimens of the original powder with varying TiC concentrations. When the TiC content is 0wt%, the microstructure of the sintered specimens is primarily lath martensite and austenite, as shown in Figure 5a,b. As shown in Figure 5c–j, the content of martensite in the specimen drops rapidly with the addition of TiC ceramics, almost no obvious martensitic organization can be detected, and the microstructure is mainly constituted of austenite. This is consistent with the results of XRD diffractograms. It can be observed through Figure 6 that Cr, Nb, and Cu elemental segregation clearly appeared at the grain boundaries of the composites. This is due to the high temperature sintering process, in which a small portion of TiC particles decomposed into Ti and C melted into the alloy, resulting in serious lattice distortion while also making some of the solid solution in the matrix solubility decrease, resulting in the production of Cr, Nb, and other compounds. The addition of TiC produces micropores and microcracks in the metallographic etching process (Figure 5d,f), which is due to a large number of defects within the lath martensite, such as dislocations, vacancies, etc., which may produce a large number of hole erosion sources during the metallographic etching process and thus are prone to etch holes and cracks.

**Figure 7:**
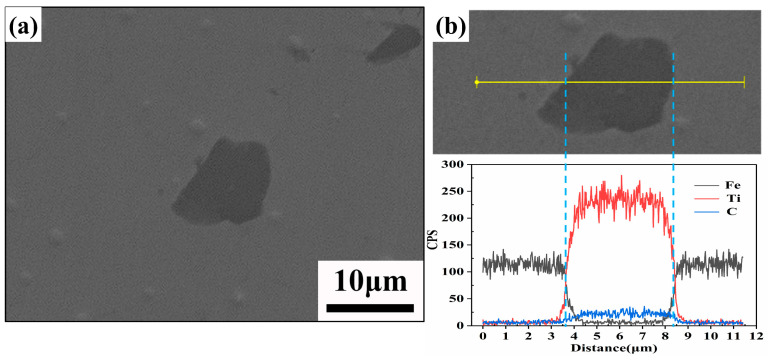
(**a**) SEM images of the non-etched TiC/17-4PH interface bonding state; (**b**) EDS maps of the non-etched TiC/17-4PH interface bonding state.

Figure 5 shows that there is a gap between the TiC particles and the matrix. However, it should be emphasized that the samples used to take the metallographic pictures were deeply etched for 5 min in order to show the microstructural features, and the gaps were caused by the etching. In fact, as shown in Figure 7a, in the non-etched sample, the TiC particles were firmly attached to the 17-4PH matrix. This is due to the fact that the wetting angle between TiC and stainless steel is only 30° at high temperatures [35], so the two materials have good bonding. Through the elemental line scan analysis of TiC and its surrounding region in Figure 7b, it can be shown that the TiC particles region is enriched with Ti and C elements, while the 17-4PH stainless steel region has fewer Ti and C elements. In the interfacial region between TiC and the 17-4PH matrix, the interdiffusion between Ti and Fe elements forms a transition layer, which indicates that good metallurgical bonding between TiC and the base metal can be obtained. The interface between TiC particles and the 17-4PH matrix is the key to fully utilizing the particle reinforcement. Compared to mechanical bonding, good metallurgical interfacial bonding helps to weaken the effect of the interface on load transfer, thus increasing the overall strength of the composite. Weak interfacial bonding, on the other hand, is prone to initiating cracking, which in turn leads to component failure under load, resulting in interfacial fracture, ceramic particle detachment, and interfacial corrosion damage, thus weakening the reinforcing effect of the ceramic particles and leading to the deterioration of the composites’ abrasion and corrosion resistance [36].

**Figure 8:**
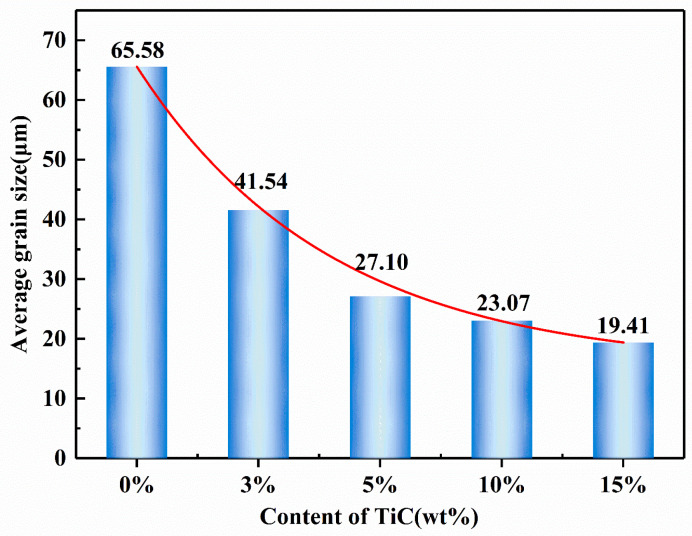
Grain size of composite materials with different TiC contents.

Figure 8 shows the average grain size of TiC/17-4PH composites with different TiC additions in Figure 5 using ImageJ (1.54 d) statistics, where the measurement method is the linear intercept method, the values are the average of five measurements, and the red curve shows the trend of the average grain size. Combined with the metallographic diagram in Figure 5a, it can be seen that the average grain size in the sintered specimen without TiC addition is relatively large, reaching 65.58 μm. Micron-TiC particles refined the grain size of the 17-4PH organization. When the TiC content continued to increase to 15 wt%, the average grain size of the 17-4PH stainless steel tissue decreased to 19.41 μm. This indicates that the incorporation of micron-TiC particles can impede the growth of dendritic crystals and form a pegging effect on the grain boundaries, significantly reducing the grain size of the matrix tissue, which can be explained by the Zener theory of grain growth [37]. In addition, when the TiC content is low, the reinforcing phases are distributed in the composites in a relatively isolated manner. With the increase in TiC content, the reinforcing phases gradually increase, interconnect with each other, and are distributed in the matrix in the form of a network, as shown in Figure 5j. The network structure can more effectively prevent the growth of matrix grains compared to individual particles [38], especially for samples with 10–15 wt% TiC added.

**Figure 9:**
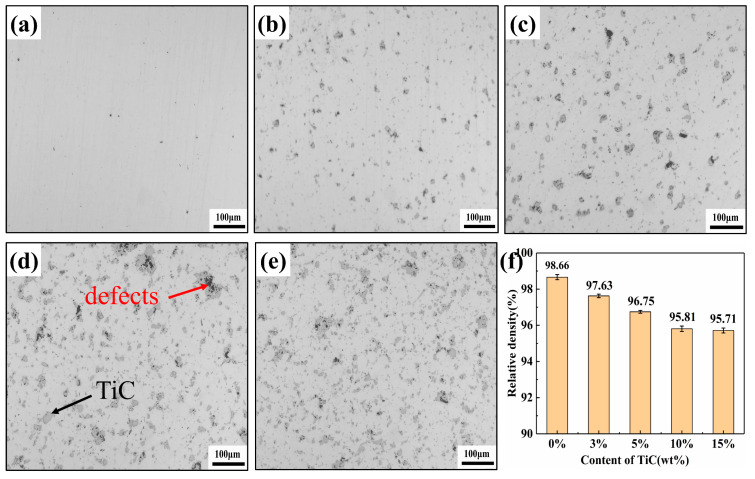
Optical micrographs showing microstructures of relative density of samples with different TiC contents: (**a**) 17-4PH stainless steel; (**b**) 17-4PH + 3 wt% TiC; (**c**) 17-4PH + 5 wt% TiC; (**d**) 17-4PH + 10 wt% TiC; (**e**) 17-4PH + 15 wt% TiC; (**f**) Relative density of samples.

Samples with dimensions of 20 × 10 × 5 mm were prepared, and the density of the specimens was measured according to Archimedes’ principle. Figure 9 illustrates the effect of TiC content on the relative densities. As shown in Figure 9f, when the TiC content is 0 wt%, the relative density can reach 98.66%. With the increase of TiC content, the relative density of the composites decreases slightly from 97.63% to 95.71%, and when the ceramic content exceeds 10wt%, the relative density of the composites is almost constant.

**Figure 10:**
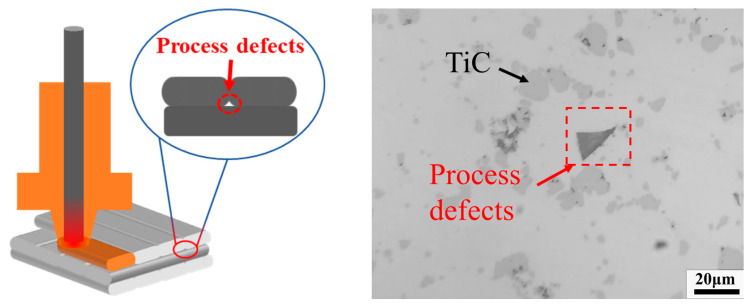
Defects formed by two adjacent low overlap rates.

The following are the main reasons for the decline in the relative density of composite materials: (1) Ceramic materials are characterized by low density, high hardness, and difficult deformation. When TiC ceramics were added, the contact area between the 17-4PH matrix and the ceramic particles increased, which led to the cutting of the matrix. This situation hindered the formation and growth of sintered necks, resulting in a gradual decrease in the relative density of the composites; (2) The holes between the agglomerated TiC particles are difficult to close during the sintering process, leading to an increase in defects in the composite, as shown in Figure 9d; (3) The irregular shape of TiC particles leads to poor material flow during 3D printing, resulting in defects in the printed specimens. Therefore, the 3D printing parameters (nozzle temperature, flow rate, layer thickness, extrusion speed, etc.) must be continuously optimized, and adjacent print channels must have a suitable overlap rate (Figure 10) to avoid defects during 3D printing.

### 3.3. Hardness and Wear Behavior

**Figure 11:**
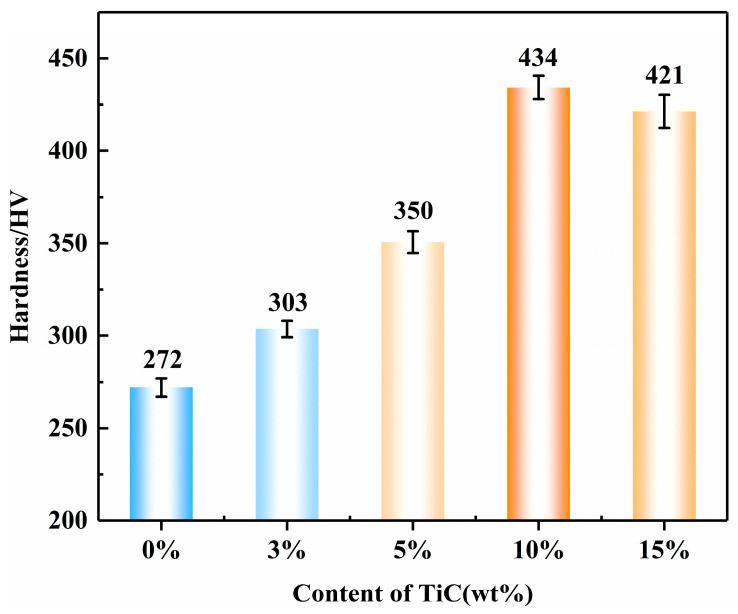
Hardness of samples with different TiC contents.

Figure 11 shows the hardness of samples with different TiC contents. Apparently, the hardness of TiC reinforced composites increased with the increase of TiC content (0–10 wt%). The 10 wt% TiC sample exhibited the highest microhardness of 434 HV. The hardness of the 15 wt% TiC samples was slightly reduced due to pore defects and TiC agglomeration in the specimens. The primary reasons for the increase in hardness after the addition of TiC are as follows: (1) Second phase strengthening: The high-hardness TiC ceramic particles (3000 HV) can inhibit the plastic deformation of the 17-4PH steel matrix and restrict the movement of dislocation, thus significantly increasing the hardness [39]; (2) Fine grain strengthening: The fine grain strengthening is generated by the addition of TiC. According to Tabor’s empirical equation (H~3σ) and the Hall–Petch equation (σ = σ_0_ + kd^−1/2^), where H and σ are the overall hardness and strength of the tested material, respectively, and d is the diameter of the grain [40]. It can be concluded that the refined microstructure leads to a significant increase in hardness; (3) The mismatch of thermomechanical properties at the interface between TiC particles and matrix can easily lead to the formation of a large number of dislocations, which produces a dislocation-strengthening effect and improves the hardness of the specimen; (4) Solid solution strengthening: The solid solution effect of Ti atoms can improve the hardness by deforming the lattice structure, clamping dislocations, and hindering dislocation movement [41].

**Figure 12:**
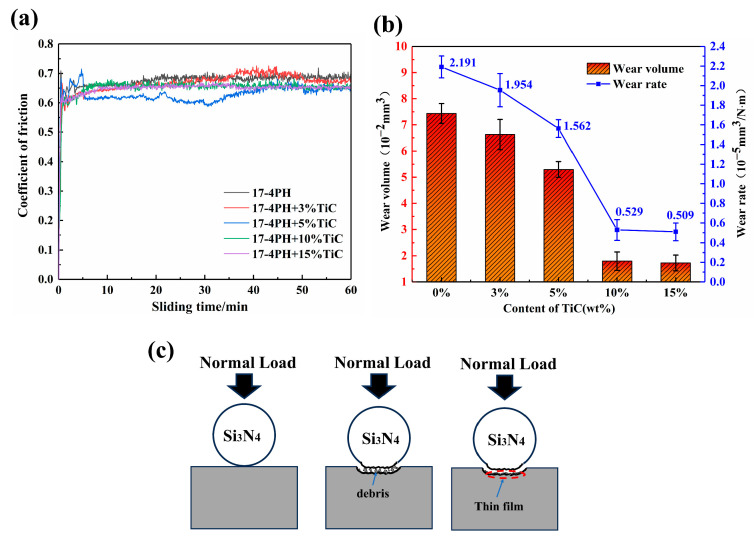
Wear properties: (**a**) coefficient of friction; (**b**) wear volume and wear rate; (**c**) Dry sliding wear process of composites.

Figure 12a shows the friction coefficient curves of different TiC contents. It can be observed that the five groups of specimens exhibit similar trends in the variation of friction coefficients. It can be clearly seen that the COF value declined with the addition of TiC. The initial low COF was due to the presence of contaminants on the mating surface. During the running-in phase, the oxide layer on the surface of the specimen was destroyed, and the coefficient of friction increased rapidly. Since the surface roughness of the specimen was not uniform, the friction coefficient appeared to fluctuate for a period of time. As the wear process proceeded, the surface roughness of the specimen tended to be consistent, and the wear process reached the stable phase [39,42].

The friction coefficient of the composite was slightly lower than that of 17-4PH stainless steel when the friction coefficient had stabilized. This was due to the formation of TiC particles in the matrix forming a uniform and continuous TiC network structure (Figure 5j) that could effectively transfer the load. This small change in COF suggests that the wear mechanism may be gradually shifting from the initial two-body wear to three-body wear, which slightly reduces the COF [43]. As the stainless steel matrix extrudes from between the TiC particles, resulting in the formation of debris between the surfaces, the TiC particles crack and break up under high Hertzian contact stresses; thus, there is a shift in the wear mechanism from two-body wear to three-body wear, with the TiC debris forming the third body. The friction layer forms through continued cyclic loading as the TiC fragments are rolled back and forth between the mating surfaces. When severe mechanical wear arises, the size of the TiC debris is significantly refined and compacted, eventually forming a uniformly thin film on the mating surface (Figure 12c) [19,44,45]. According to Figure 12b, the wear rate of 17-4PH stainless steel reduced from 2.191 × 10^−5^ mm^3^ /(N‧m) to 0.509 × 10^−5^ mm^3^ /(N‧m) following the addition of TiC, and the wear resistance increased dramatically. Interestingly, the hardness of the 15 wt% TiC composites was significantly higher than that of the 10 wt% TiC composites, but there was no significant reduction in the wear rate, which was 0.529 × 10^−5^ mm^3^ /(N‧m) and 0.509 × 10^−5^ mm^3^ /(N‧m) for the composites with 15 wt% TiC and 10 wt% TiC, respectively. This phenomenon seems to violate Archard’s law [46]. Due to the defects in the 15 wt% TiC/17-4PH composites, part of the TiC particles peeled off during wear, which negatively affected the wear resistance.

**Figure 13:**
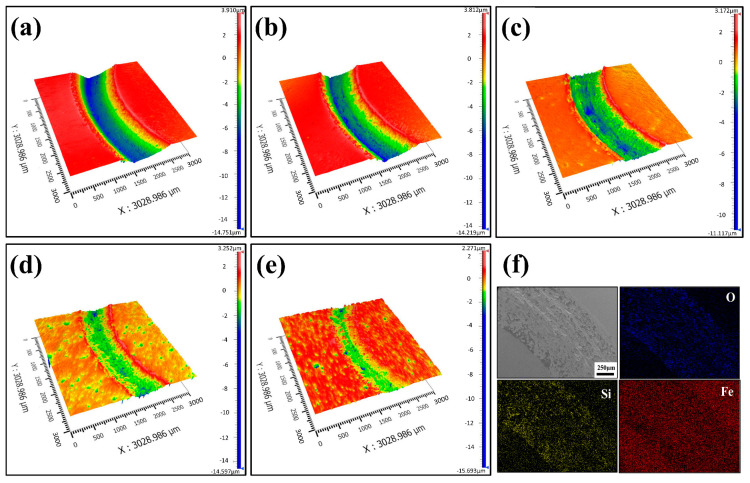
3D wear morphology with different TiC contents: (**a**) 17-4PH stainless steel; (**b**) 17-4PH + 3 wt% TiC; (**c**) 17-4PH + 5 wt% TiC; (**d**) 17-4PH + 10 wt% TiC; (**e**) 17-4PH + 15 wt% TiC; (**f**) EDS analysis of the wear tracks.

The 3D wear morphology of composite specimens with different contents of TiC is shown in Figure 13. The comparison indicated that the wear surface of 17-4PH stainless steel had obvious grooves, and the width of the wear marks was the largest. With the increase of the mass fraction of TiC particles, the grooves on the wear surface of the TiC/17-4PH composite specimens were gradually reduced, and the width of the wear marks and the depth of the wear were significantly reduced. Therefore, the addition of TiC particles to the 17-4PH stainless steel matrix effectively reduced the wear rate of the composites and improved the wear resistance, which is consistent with the change in wear rate in previous studies. From Figure 13f, it can be concluded that the O element content at the wear marks was relatively high. This is due to the fact that during the high-speed wear process of TiC/17-4PH composite, the temperature of the wear surface increases instantly, and the O_2_ in the air easily reacts with Fe to form an oxide film, which reduces the friction coefficient of the composite and thus reduces the wear of the material [43].

**Figure 14:**
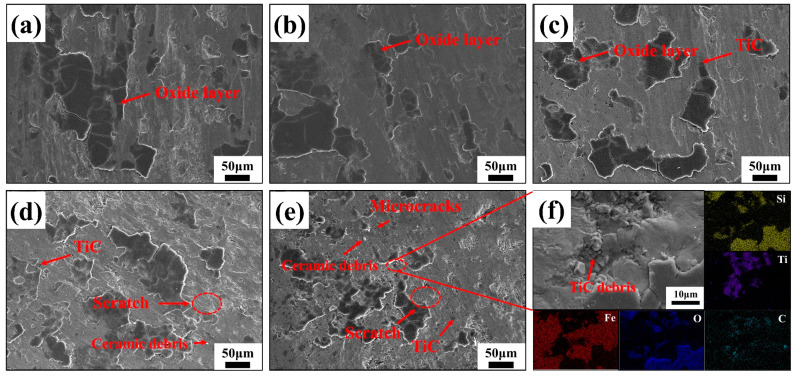
Surface wear morphology with different TiC contents: (**a**) 17-4PH stainless steel; (**b**) 17-4PH + 3 wt% TiC; (**c**) 17-4PH + 5 wt% TiC; (**d**) 17-4PH + 10 wt% TiC; (**e**) 17-4PH + 15 wt% TiC; (**f**) EDS analysis of the wear surfaces.

To further reveal the wear mechanism, the surface micro-morphology of the wear marks was analyzed, as shown in Figure 14. Without the addition of TiC particles, the matrix’s hardness was relatively low, which resulted in severe shear resistance and plow resistance between the substrate and the silicon nitride balls. In this situation, the abrasion marks’ surface displayed clear adhesion marks, plenty of laminar fatigue flaking, and minor scratches [39,45]. The main wear mechanisms for the 17-4PH stainless steel matrix were severe plastic deformation and adhesive wear. Plastic deformation and layer spalling significantly reduced when 3 wt% TiC particles were added, indicating that a small amount of TiC particles counteracted part of the shear resistance, thus preventing the extrusion deformation of the Si_3_N_4_ grinding balls on the substrate and reducing the spalling of the plastic stacked layer.

In contrast, obvious adhesion marks, slight plastic deformation, and scratches were observed on the wear surface of 5 wt% TiC/17-4PH (Figure 14c), where the TiC particles clearly hindered the plastic deformation and shear of the matrix, indicating that the wear resistance of the composites has been significantly improved compared to that of 17-4PH stainless steel. However, A few TiC fragments caused minor scratches on the mating surface. The wear mechanism of 5 wt% TiC/17-4PH consisted mainly of minor abrasive wear and adhesive wear.

**Figure 15:**
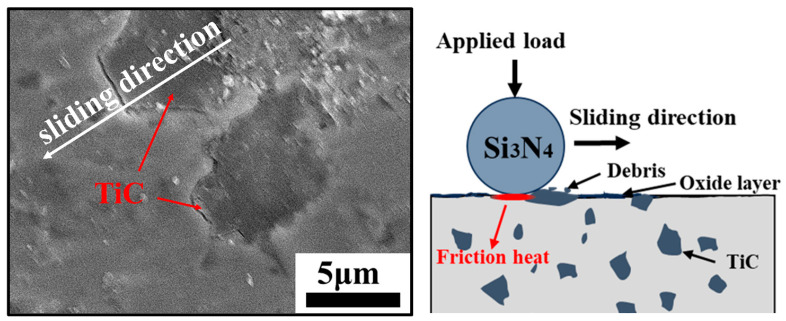
The schematic diagram of dry sliding wear of TiC/17-4PH composite material.

The deformation of the mating surfaces of 10 wt% TiC/17-4PH became shallower relative to 5 wt% TiC. It is clear that the wear resistance of the composites was greatly improved with the increase of TiC content. TiC particles play a crucial role in the wear process, as they impede plastic deformation and shearing force in 17-4PH stainless steel (Figure 15) [47]. However, TiC particles rupture under high Hertzian contact stresses. The spalling of TiC debris generates a new abrasive between the two grinding parts, resulting in significant scratches on the mating surfaces [19,39]. Overall, the main wear mechanisms for 10 wt% TiC/17-4PH were slight abrasive wear and adhesive wear. When the TiC content was increased to 15 wt%, the plastic buildup on the mating surface was significantly reduced, but microcracks and abrasive particles were present on the wear surface (Figure 14e). Microcracks occurred due to the stress concentration caused by the TiC particles in the steel matrix during the wear process, and cracks generated by stress concentration were characteristic of fatigue wear. From Figure 14f, it can be seen that the appearance of abrasive particles was due to the increase in defects caused by TiC aggregation on the surface of the composite. During the high-speed wear process, the TiC particles at the edges of the defects were broken, torn, and detached from the 17-4PH matrix to form abrasive particles [48]. The exfoliated large TiC particles worked together with Si_3_N_4_ grinding balls to intensify the degree of wear [49]. Therefore, the improvement of wear resistance was limited with 15 wt% TiC compared to composites with 10wt% TiC added. The main wear mechanisms of 15 wt% TiC/17-4PH composites were abrasive wear, fatigue wear, and adhesive wear.

In conclusion, the wear resistance of TiC-reinforced 17-4PH composites increased significantly with the increase of TiC content (3–15 wt%), and the wear mechanism changed from plastic deformation and adhesive wear to abrasive wear and adhesive wear. Because of the low hardness of 17-4PH stainless steel, the shear force generated during wear was higher than the strength of the metal, resulting in severe plastic deformation and significant adhesive wear on the wear surface. The incorporation of TiC improved the hardness of the reinforced composites under the same wear conditions and protected the matrix during the wear process, thus reducing the friction coefficient and volumetric wear rate. However, compared to 10 wt% TiC composites, 15 wt% TiC composites have limited improvement in wear resistance due to pore defects and TiC agglomeration.

## 4. Conclusions

In this paper, micron-TiC-particle-reinforced 17-4PH stainless steel composites were prepared by the metal fused filament fabrication technique. The effect of TiC particles with different contents on the organization and properties of 17-4PH stainless steel was studied, and the following conclusions were obtained:

1. TiC-reinforced 17-4PH stainless steel composites with high densities and excellent wear resistance can be prepared by fused filament fabrication. The TiC contents were 0, 3, 5, 10, and 15 wt%. The TiC particles were uniformly distributed in the composites prepared by this technology.
2. Compared with the samples without TiC, the addition of micron-TiC significantly refined the grains. The composite material primarily consisted of austenite and a small amount of martensitic phase content. The addition of TiC caused a slight decrease in the relative densities of the materials, but both remained above 95%. The change in the relative density of the composites leveled off when the ceramic content exceeded 10 wt%.
3. The bonding method between TiC particles and the 17-4PH matrix was metallurgical bonding. When the TiC content was 10 wt%, the hardness of the composites increased from 272 HV to 434 HV, an increase of 59.56%. The hardness of the 15 wt% TiC samples was slightly reduced due to pore defects and TiC agglomeration in the specimens. The hardness enhancement can be attributed to fine grain strengthening, solid solution strengthening, and second phase strengthening.
4. The addition of TiC particles significantly improved the wear resistance of the 17-4PH matrix, reducing the wear rate from 2.191 × 10^−5^ mm^3^ /(N‧m) to 0.509 × 10^−5^ mm^3^/(N‧m), which is a 3.3-fold increase in wear resistance. The wear mechanisms of composites were mainly plastic deformation, adhesive wear, and abrasive wear. Compared to 10 wt% TiC composites, 15 wt% TiC composites had limited improvement in wear resistance due to microcracks and TiC agglomeration.

## Figures and Tables

**Table 1:** Composition of 17-4PH stainless steel (wt%).

Elements	Cr	Ni	Cu	Nb	Mn	Si	C	Fe
Content	16.30	4.25	3.85	0.22	0.53	0.45	0.034	Bal.

**Table 2:** 3D printing conditions.

Printing Parameter	Value	Unit	Printing Parameter	Value	Unit
Nozzle Diameter	0.4	mm	Infill Density	100	%
Layer Height	0.1	mm	Infill Overlap	35	%
Nozzle Temperature	245	℃	Printing Rate	50	mm/s
Heated Bed Temperature	100	℃	Infill Pattern Type	Spiral Icositetrahedron	_

## Data Availability

The data presented in this study are available on request from the corresponding author.
